# Molecular Functions of WWOX Potentially Involved in Cancer Development

**DOI:** 10.3390/cells10051051

**Published:** 2021-04-29

**Authors:** Karim Taouis, Keltouma Driouch, Rosette Lidereau, François Lallemand

**Affiliations:** Pharmacogenomics Unit, Department of Genetics, Institut Curie, 75005 Paris, France; karim.taouis@curie.fr (K.T.); keltouma.driouch@curie.fr (K.D.); rosette.lidereau@curie.fr (R.L.)

**Keywords:** WWOX, cancers, molecular functions

## Abstract

The WW domain-containing oxidoreductase gene (*WWOX*) was cloned 21 years ago as a putative tumor suppressor gene mapping to chromosomal fragile site FRA16D. The localization of *WWOX* in a chromosomal region frequently altered in human cancers has initiated multiple current studies to establish its role in this disease. All of this work suggests that WWOX, due to its ability to interact with a large number of partners, exerts its tumor suppressive activity through a wide variety of molecular actions that are mostly cell specific.

## 1. Introduction

The WW domain-containing oxidoreductase gene (*WWOX*) was initially identified as a gene present in the chromosomal region 16q23.3-24.1 frequently affected by loss of heterozygosity (LOH) in various cancers [1,2,3,4,5,6]. Homozygous deletions of *WWOX* have also been detected in different cancer cells [7]. This gene, consisting of 1.1 million bases, is composed of 9 exons; its intron 8 contains the common fragile site FRA16D which would be the major cause of the instability of the 16q23.3-24.1 region [6]. The localization of *WWOX* in this unstable chromosomal region was the first argument suggesting that it is a tumor suppressor gene. As a result, many researchers have set out to study the possible association of WWOX with cancer.

*WWOX* encodes a protein of 414 amino acids composed of a short-chain dehydrogenase/reductase central domain (SDR domain) that may be involved in sex-steroid metabolism and two NH2-terminus WW domains (WW1 and WW2) responsible for multiple protein-protein interactions [1,8,9,10]. So far, more than 570 interactors of WWOX have been identified [8,11] (https://thebiogrid.org/119707/summary/homo-sapiens/wwox.html, accessed date: 22 March 2021). Over time, numerous observations in the clinical setting and experimental results supporting the assumption that WWOX plays a critical role in cancer have accumulated.

First, in human tumors, *WWOX* expression has been shown to be decreased or absent in several types of cancer [12]. This low expression is associated with a poor prognosis in breast [13,14,15,16,17,18,19], ovarian [20], bladder [21], liver [22,23], and kidney cancers [24]. Immunohistochemistry studies have shown that WWOX protein levels can vary depending on tumor subtypes. In breast cancer, for instance, WWOX is more highly expressed in luminal tumors since *WWOX* is an estrogen-regulated gene. Loss of WWOX expression is associated with a poor prognosis in ER-negative breast tumors but not as much in ER-positive breast cancers [1,25,26]. Genomic losses appear to be the main mechanism leading to inhibition of WWOX expression. Thus, more than half of breast and ovarian cancers have heterozygous deletions within the *WWOX* gene [25]. WWOX expression is also inhibited by its hypermethylation in some cancer cells [26], and the tyrosine kinase Ack-1 phosphorylates WWOX on its tyrosine 287, which induces its polyubiquitination and thereby its degradation by the proteasome in prostate cancer [27]. However, *WWOX* mutations are rare events in cancers. It has been proposed that due to its localization in an unstable genomic region, *WWOX* suppressor activity is much more frequently inhibited by LOH and homozygous deletion than by point mutations [28]. Importantly, germline inactivation *WWOX* gene has been described in humans as leading to autosomal recessive disorders such as WOREE syndrome (WWOX-related epileptic encephalopathy). Even though homozygous deletions/mutations completely inactivating the WWOX gene are rare, neither the hundreds of patients reported worldwide nor the parents of these children, who by definition are WWOX haploinsufficient, developed neoplasia. Thus the role of WWOX in cancer is probably more prominent as a relevant gene in tumor progression rather than tumor initiation [29,30].

Secondly, the ability of WWOX to inhibit proliferation, support-dependent growth, migration, invasion, tumor growth, and metastasis of many different cell lines has been reported, which is consistent with tumor suppressive activity [15,17,26,31,32,33,34,35]. Moreover, different groups of researchers have defined WWOX as a proapoptotic protein, raising the possibility that WWOX may inhibit carcinogenesis by inducing cell death [3,14,32,36,37,38,39,40].

Thirdly, mouse models also shed light on the tumor suppressor activity of WWOX. Different models of mice carrying a targeted deletion of the *Wwox* gene have been generated. In a B6-129 mixed background, juvenile *Wwox−/−* mice exhibited more osteosarcomas than wild-type littermates, while more lung papillary carcinoma appeared in adult *Wwox+/−* mice in comparison with *Wwox+/+* mice [41]. Heterozygous *Wwox+/−* mice also showed a higher incidence of ethyl nitrosourea-induced lung tumors and lymphomas than control mice. Interestingly, tumors from *Wwox+/−* mice expressed a low level of Wwox protein, suggesting that haploinsufficiency of *WWOX* could promote carcinogenesis. Consistent with this hypothesis, Abdeen et al. showed that the *Wwox+/−* tumor-susceptible C3H mice had a higher frequency of mammary tumors than control mice [42]. Ludes-Meyers et al. reported that a *Wwox* hypomorphic female mice had a higher incidence of spontaneous B-cell lymphomas [43]. However, it should be noted that other groups have also generated *Wwox* knockout mice showing no spontaneous neoplasia [44,45]. In particular, Chou et al. have recently shown that skin development is impaired in *Wwox−/−* mice but they have not described any tumor [46]. Moreover, Suzuki et al. did not report any tumors in the lde/lde rats which have spontaneous homozygous inactivation of the *Wwox* locus [47]. The reasons for these discrepancies remain to be determined. One possibility might be that the Wwox KO mice have extremely fragile bones. Frequent fractures can occur in newborn mice and the originally diagnosed periosteal osteosarcomas might be misdiagnoses. Since knockout mice led to early postnatal lethality, conditional *Wwox* deletion, by means of Cre recombinase approaches have been performed in different mice tissues. The study of these animal models has shown that the ablation of *Wwox* in the epithelium of the mammary glands of C3H mice or in the liver of C57BL6/j;129v mice treated with diethylnitrosamine, increases tumorigenesis [22,48]. However, *Wwox* ablation in the mammary gland, the bones (osteoblasts), or the liver in strains other than C3H mice has been shown to be non-carcinogenic. WWOX loss would therefore contribute to the progression of carcinogenesis rather than the initiation of the tumor [22,49,50,51].

Fourth, Chang et al. reported that different peptides of WWOX (WWOX286-299, WWOX7-21, and WWOX7-11) injected into the tail vein of mice inhibit the growth of melanoma B16F12 cells inoculated into the flanks, revealing their potential use in anticancer treatments [52,53]. Additionally, the WWOX7-21 peptide promotes ceritinib-mediated 4T1 breast-cancer stem cells apoptosis [53]. The authors assume that these peptides mimic the anticancer function of endogenous WWOX.

Fifth, the same group demonstrated that the WWOX-deficient metastatic cancer cells escape from WWOX-expressing cells by retrograde migration and by inducing apoptosis of the latter [54]. These results strongly suggest that the WWOX-deficient cells must suppress WWOX-expressing cells to metastasize.

The extensive study of the molecular functions of WWOX has provided many other arguments, leaving little doubt that WWOX plays an important role in cancer, which would therefore be associated with tumor progression. In this review, we discuss some molecular functions of WWOX, which may explain its anticarcinogenic activity. In particular, we described the main molecular partners of WWOX involved in these molecular functions (Table 1).

## 2. WWOX a Positive Regulator of Cell Death

Different groups of researchers have highlighted that WWOX induces cell death via multiple cell-specific molecular mechanisms. The inhibition of WWOX expression would therefore make cancer cells resistant to cell death, which is favorable to the development of cancer.

Chang et al. were the first to define WWOX as a proapoptotic protein raising the possibility that WWOX might inhibit carcinogenesis by inducing this type of cell death [3]. WWOX is crucial for various stress stimuli, such as TNF (tumor necrosis factor), UV (ultraviolet) light, and ectopic expression of the proapoptotic protein p53, to mediate cell death in different cell lines [37]. In response to stimuli such as UV light, WWOX is phosphorylated on its tyrosine 33 located in the WW1 domain (pY33-WWOX). This phosphorylation promotes the WWOX-p53 association and the translocation of the complex to the nucleus. WWOX increases the stabilization of p53. Mutant forms of WWOX, unable to translocate to the nucleus or to be phosphorylated on their tyrosine 33, are less effective at inducing apoptosis than wild-type WWOX [36]. Altogether, these observations indicate that WWOX is a proapoptotic protein acting by binding p53 in the nucleus. However, it has also been reported that WWOX induces apoptosis in p53-deficient human lung cancer NCI-H1299 cells, thus WWOX is also able to stimulate programmed cell death independently of p53 [3].

In agreement with this last observation, by using two-hybrid and co-immunoprecipitation analyses, the same group have highlighted the interactions of WWOX with TRADD (tumor necrosis factor receptor type 1) and TRAF2 (TNF receptor-associated factor 2), two components of the TNF signaling pathways, suggesting that WWOX may modulate TNF-dependent cell death by regulating the function of these two proteins in TNF signaling [55].

In addition, sciatic nerve transection in rat induces (1) expression of WWOX; (2) nuclear translocation of pY33-WWOX with several proteins involved in transcription: p-CREB (cyclic AMP responsive element binding protein 1), JNK (c-Jun amino-terminal kinase), c-Jun (AP-1 transcription factor subunit), NF-κB (nuclear factor kappa B subunit 1), and ATF3 (activating transcription factor 3); (3) the pY33-WWOX-p-CREB interaction; and then (4) apoptosis. Moreover, CREB enhances the apoptotic function of WWOX in human neuroblastoma SK-N-SH cells. These results suggest that WWOX induces apoptosis in neurons by interacting with CREB in the nucleus [56].

They have also determined that the cytokine TGF-β1 (transforming growth factor β1) binds to Hyal-2 (hyaluronidase 2) located on the cell surface. The binding of TGF-β1 to Hyal-2 induces the pY33-WWOX-Hyal-2 interaction and the translocation of the resulting complex to the nucleus. This complex then activates SMAD (mothers against decapentaplegic homolog)-dependent transcription and apoptosis in human colon HCT116 cells [57,58]. In addition, HA (hyaluronan), one of the main components of the extracellular matrix, induces the nuclear translocation of WWOX, Hyal-2 and SMAD4 (mothers against decapentaplegic homolog 4) in several cell lines [57]. HA induces WWOX-Hyal-2-SMAD4 complex formation and bubbling cell death in human prostate cancer DU145 cells. By interacting with SMAD4 and Hyal-2, WWOX would therefore be able to induce different types of cell death [57].

In parallel, Aqeilan et al. reported that the WW1 domain of WWOX binds to the PPPY487 motif of the proapoptotic protein p73 (a p53 homolog) in human embryonic kidney HEK293 cells [38]. The phosphorylation of WWOX on its tyrosine 33 by the SRC kinase, enhances this interaction. WWOX sequesters p73 in the cytoplasm (in NIH3T3 mouse fibroblasts and human breast MCF7 cells), and cytoplasmic p73 contributes to the proapoptotic activity of WWOX (in human osteosarcoma SAOS-2 cells). These results suggest that the WWOX-p73 cytoplasmic complex would induce apoptosis in specific cells.

**Figure 1:**
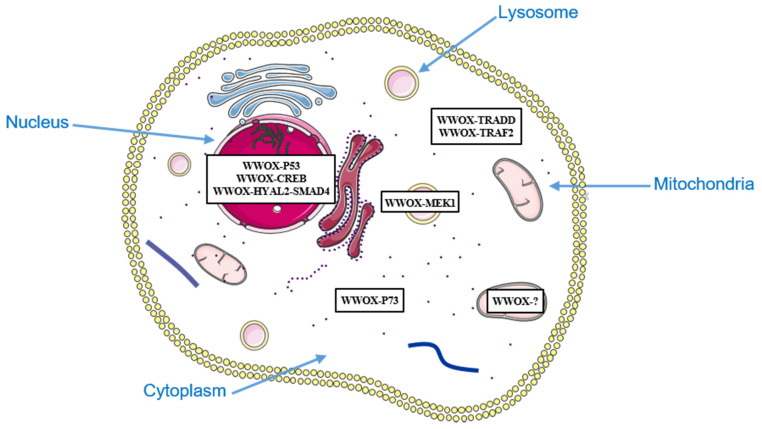
Subcellular localization of WWOX and its partners involved in cell death.

Another group has shown that a dominant-negative form of WWOX or WWOX knockdown can inhibit apoptosis induced by PMA (phorbol 12-myristate 13-acetate) in human Jurkat T cells. PMA-induced apoptosis is associated with dissociation of the WWOX-MEK1 (mitogen-activated protein kinase kinase 1) complex located in lysosomes, and translocation of WWOX into mitochondria. WWOX could therefore induce mitochondrial apoptosis in Jurkat T cells [59] (Figure 1).

## 3. WWOX Associates with Cancer-Linked Transcription Factors and Inhibits Their Transcriptional Activity

WWOW has been clearly shown to negatively modulate various oncogenic transcription factors. The inhibition of the expression of WWOX could therefore induce an over activity of the latter, thus enhancing carcinogenesis.

The AP-2 (transcription factor AP-2) family is composed of five transcription factors (AP-2α, AP-2β, AP-2γ, AP-2δ, and AP-2ε) [71]. AP-2γ exhibits the characteristics of oncogenes or tumor suppressor genes depending on the type of cancerous tissue. It is overexpressed in invasive breast cancer and triple-negative breast tumor (absence of estrogen and progesterone receptor expressions, and overexpression of ERBB2 (erb-b2 receptor tyrosine kinase 4)). This overexpression is associated with a poorer response to endocrine therapy and with reduced patient survival [16,72,73,74]. WWOX interacts with AP-2 γ and, to a lesser extent, AP-2α [62]. The WW1 domain of WWOX and the PPPYFPPPY64 motif of AP-2γ are involved in this interaction. Using NIH3T3 mouse fibroblasts and human cervical cancer Hela cells as cell models, ectopic expression of WWOX has been shown to inhibit the transcriptional activity of AP-2γ by sequestrating it into the cytoplasmic compartment.

Human epidermal growth factor receptor 1 (EGFR/ERBB1), ERBB2, ERBB3, and ERBB4 are members of the epidermal growth factor receptor subfamily of the receptor tyrosine kinases family [75]. Activation of ERBB4 leads to the stimulation of the Ras/mitogen activated protein kinase (MAPK) and phosphoinositide 3-kinase (PI3-K)/Akt pathways, and to the release of its intracellular domain (CTF) in the cytoplasm. CTF then moves to the nucleus where it functions as a coactivator or a corepressor for different transcription factors such as yes-associated protein (YAP). The role of ERBB4 in cancer is still unclear. Series of experimental studies suggest that it has both oncogenic and tumor suppressive functions in this cancer. With the HEK293 and Hela cells as cell models, the WW1 domain of WWOX has been reported to bind to two PPxY motifs of ERBB4 (PPIY1037 and PPPY1285) [63]. WWOX competes with YAP for CTF and sequesters CTF in the cytoplasm, thereby inhibiting the transcriptional activity of the CTF-YAP complex.

AP-1 (Activator protein-1) designates a family of oncogenic transcription factors whose activity is modulated by numerous intracellular signaling pathways controlling differentiation, migration, proliferation, and apoptosis [76]. AP-1 functions as homo- or hetero-dimeric combinations of proteins of the JUN and FOS sub-families. Different observations suggest that C-JUN, a member of the JUN sub-family, plays an important role in various types of cancers. Indeed, this protein is overexpressed in several cancers including breast, colorectal, and lung cancers. Moreover, overexpression of C-JUN in the breast cell line MCF7 has been shown to stimulate a tumorigenic and invasive phenotype. WWOX, via its WW1 domain, binds the PPPY64 motif of C-JUN and sequesters it in the cytoplasm, thereby inhibiting its transcriptional activity [64].

RUNX2 (Runt-related transcription factor 2), belonging to the RUNX family, is an oncogenic transcription factor that interacts with multiple cell-specific co-regulators to modulate gene expression [77,78]. It plays an important role in breast, ovarian, prostate, and other cancers. In breast cancer, RUNX2 is crucial for invasion and bone metastasis [79,80]. WWOX interacts with RUNX2 via its WW1 domain and inhibits its transcriptional activity in various cell types [65]. WWOX binds to RUNX2 located on the osteocalcin promoter and inhibits its positive effect on the activity of this promoter in the mouse osteoblast MC3T3 cells. Therefore, WWOX does not inhibit the transcriptional activity of RUNX2 by sequestrating it in the cytoplasm but rather as a transcriptional corepressor. Ectopic expression of WWOX in different osteosarcoma cell lines inhibits metastasis, and the expression of *RUNX2* and its target genes such as *VEGF* (vascular endothelial growth factor gene) involved in angiogenesis and *MMP13* (matrix metalloproteinase-13 gene) involved in invasion [81]. Zheng’s study strongly suggests that WWOX inhibits invasion of lung cancer cells by inhibiting RUNX2 expression [82].

## 4. WWOX Interacts and Affects the Function of Components of Oncogenic Pathways

Various studies have provided experimental arguments indicating that WWOX is a negative regulator of the oncogenic pathways: Wnt (Wingless)/β-catenin, TGFβ1/SMAD and JAK2 (Janus kinase 2)/STAT3 (signal transducer and activator of transcription 3) signaling pathways. The inhibition of the expression of WWOX would increase their activity to an aberrant level, thus promoting the development of many types of cancer.

The Wnt/β-catein pathway modulates proliferation, differentiation, apoptosis, migration, invasion, and tissue homeostasis; its aberrant activation stimulates the development of cancers including colon and breast cancers [83,84,85]. This pathway regulates the transcriptional activity of Tcf (T-cell factor) and Lef (lymphoid enhancer factors) by modulating the stability of β-catenin [86]. In the absence of secreted Wnt ligand, GSK3β (glycogen synthase kinase3β) and CK1α (casein kinase 1α) integrated in the degradation complex of β-catenin, phosphorylate β-catenin, this induces its ubiquitination and therefore its degradation by the 26S proteasome. The association of the Wnt ligand with its receptors (Frizzled and low-density lipoprotein receptor-related protein) inhibits the phosphorylation of β-catenin by GSK3β and CK1α, which induces its accumulation in the nucleus. In the nuclear compartment, β-catenin associates with Tcf and Lef and stimulates their transcriptional activity thus activating the expression of target genes. BCL9-2 (B-cell CLL/lymphoma 9-like protein), a component of the BCL9 family of vertebrate, is a nuclear coactivator of β-catenin in the Wnt/β-catenin pathway [87]. Dishevelled proteins1, 2 and 3 (Dvl-1, 2, and 3) are crucial for the Wnt/β-catein pathway to stabilize β-catenin and the β-catenin-Tcf interaction [88,89]. Nuclear translocation of Dvl proteins is required for their function in Wnt/β-catenin signaling [88]. WWOX has been defined as a novel inhibitor of the Wnt/β-catenin pathway [60,61]. In HEK293 and the breast cell line MCF7 as cellular models, WWOX has been shown to interact with Dvl and BCL9-2 proteins [8,60,61,90]. The WW1 domain of WWOX, the PPPY568 motif of Dvl-2, and the PPPY561 motif of BCL9-2 play important roles in these interactions. It has been suggested that WWOX inhibits this pathway at least in part by (1) sequestrating Dvl-2 in the cytoplasm and (2) binding BCL9-2 in the nucleus and suppressing its transcriptional activity.

The cytokine TGFβ1 modulates multiple cellular processes such as proliferation, differentiation, and apoptosis, and thus plays a crucial role in embryogenesis and homeostasis of adult tissues [91]. TGFβ1 acts by activating several intracellular signaling pathways, including Erk (extracellular signal-regulated kinase), JNK, p38 mitogen-activated protein kinase, PI3K (phosphoinositide 3-kinase), SMAD, and other pathways. Regarding the SMAD pathway, the binding of TGFβ1 to its receptors (TGFβ type I (TβRI) and TGFβ type II (TβRII)) induces the phosphorylation of SMAD3 (mothers against decapentaplegic homolog 3) by TβRI. This phosphorylation induces the association of SMAD3 with SMAD4 and the translocation of the resulting complex to the nucleus where it modulates the transcription of targets genes. TGFβ1 plays an intricate role in cancer. It has tumor suppressive activity during the first stages of carcinogenesis but promotes cancer development in the later stages. Ferguson and collaborators have shown that WWOX inhibits the transcriptional activity of TGFβ1 in the breast cell line MCF10 [34]. WWOX, via its WW1 domain, binds SMAD3 and sequesters it to the cytoplasm. WWOX would therefore inhibit the TGFβ1/SMAD pathway at least in part by sequestrating SMAD3 in the cytoplasm.

**Figure 2:**
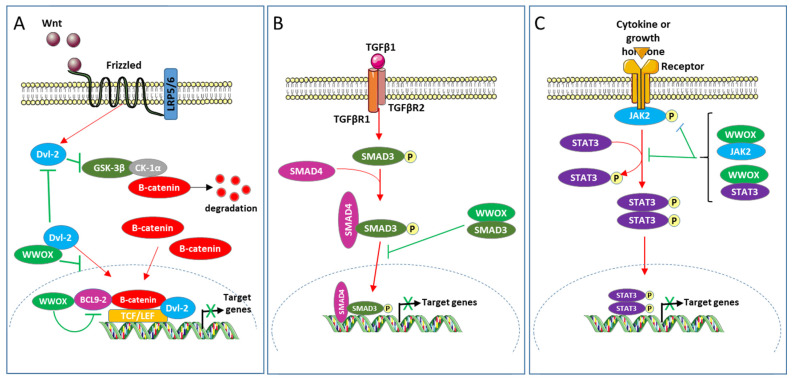
(**A**) Modulation of Wnt/β-catenin, (**B**) TGFβ1 and (**C**) JAK2/STAT3 pathways by WWOX.

The JAK2/STAT3 pathway consists of the intracellular protein tyrosine kinase JAK2 and the transcription factor STAT3 [92]. This pathway mediates the signaling of various cytokines, growth factors, and hormones. The association of JAK2 with the intracellular part of its receptor induces the phosphorylation of STAT3 by JAK2, leading to the translocation of STAT3 in the nucleus where it stimulates the transcription of target genes. The JAK2/STAT3 signaling pathway is aberrantly activated in various tumors types, which is generally associated with poor prognosis [93]. Chang and collaborators showed that WWOX interacts with JAK2 and STAT3 via its WW1 domain. WWOX prevents phosphorylation of JAK2 and STAT3, and inhibits the transcriptional activity of STAT3 in different TNBC (triple-negative breast cancer) cell lines [15]. Expression of WWOX and phosphorylated STAT3 are negatively correlated in breast cancer cells. Ectopic expression of WWOX inhibits migration, tumor proliferation and metastasis of TNBC cell lines MDA-MB-231. This ectopic expression also inhibits the migration and growth of cells induced by STAT3 in NIH3T3. These results strongly suggest that the loss of WWOX drives metastasis in TNBC by JAK2/STAT3 axis [15] (Figure 2).

Ezrin is an actin-binding protein belonging to the ERM (Ezrin/Radixin/Moesin) family of proteins that regulate cell polarity, adhesion, and invasion through the control of various signaling pathways [94]. Ezrin has been shown to promote cell migration and invasion in several types of cancer where high protein expression has been correlated with poor patient prognosis. The interaction of Ezrin and WWOX has been revealed in gastric parietal cells. PKA-mediated phosphorylation of Ezrin regulates Ezrin–WWOX interaction and WWOX targeting to apical membranes of cells. The Ezrin-WWOX complex may play a role in the remodeling of the apical membrane cytoskeleton and the recruitment of the pump of proton H,K-ATPase to apical membrane upon activation of the parietal cell [68]. The role of the WWOX-Ezrin complex in cancer has yet to be determined.

## 5. WWOX, the Hypoxia-Inducible Transcription Factor 1α (HIF1α) and the Warburg Effect

Most tumor cells need to synthesize their ATP mainly via anaerobic glycolysis (Warburg effect) to proliferate under hypoxic conditions and generate glycolytic intermediates involved in the biosynthesis of macromolecules and organelles essential for assembling new cells [95]. HIF1α, overexpressed in common human cancers, is essential to this cellular adaptation [96]. This transcription factor induces glycolysis by upregulating the expression of various genes of the glycolytic pathway (hexokinase 1 and 2, glucose transporters 1 and 3, lactate dehydrogenase-A), and restricts mitochondrial respiration by stimulating pyruvate dehydrogenase kinase-1.

Inactivation of the *WWOX* gene in mouse embryonic fibroblasts (KO-MEFs) increases the glucose uptake, improves glycolysis, reduces mitochondrial respiration, stimulates expression of HIF1α target genes, and promotes tumorigenicity [97]. Inhibition of HIF1α expression prevents increased of glucose uptake and tumorigenicity in the KO-MEFs [97]. Using WWOX liver-specific knockout mice, it has also been reported that inhibition of HIF1α significantly delays hepatocellular carcinoma induced by loss of WWOX expression [22]. WWOX, via its WW1 domain, interacts with HIF1α and induces its degradation [97].

**Figure 3:**
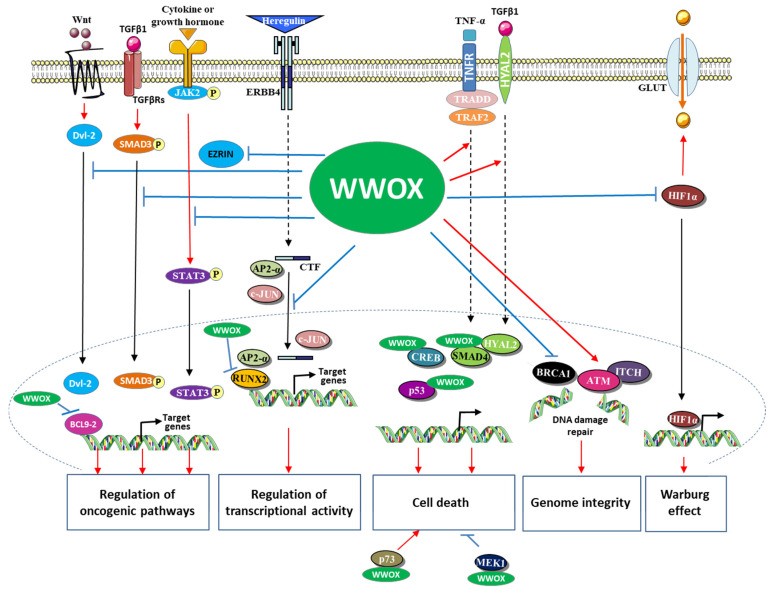
Main anticarcinogen molecular functions of WWOX.

These results strongly suggest that the loss of expression of WWOX would induce the expression of HIF1α, thus promoting anaerobic glycolysis and therefore cancer (Figure 3).

## 6. WWOX, a Genome Stability Gatekeeper

The maintenance of genome stability is essential to cell survival and the elimination of DNA disorders that can cause cancer. To maintain their genome integrity, cells have developed many molecular mechanisms to repair their DNA damages [98,99,100].

DNA double-strand breaks (DSBs) are one of the most dangerous forms of DNA damage [98]. Two major molecular mechanisms are involved in DSB repair: NHEJ (Non homologous end joining) and HR (homologous recombination) [101]. NHEJ, active throughout the cell cycle, joins broken ends by religation. HR, considered to be more faithful than NHEJ, induces recombination between sister chromatids during the S and G2 phases of the cell cycle. The MRE11 (Meiotic recombinase 11)-RAD50-NBS1 (Nijmegen Breakage Syndrome 1) complex initiates repair of DSBs by recognizing and binding them. This complex then recruits and activates the ATM protein kinase. ATM (ataxia telangiectasia mutated) induces NHEJ or HR, depending on the intracellular context and the location of the DSBs [102,103]. To induce HR, ATM phosphorylates many substrates such as BRCA1 (breast cancer type 1) and CtIP (DNA endonuclease RBBP8). These substrates promote the resection of the DNA ends on either side of the DNA break to generate single stranded ends that will be coated by RPA (replication protein A) then by RAD51(recombinase RAD51). The resulting nucleoprotein filaments invade the complementary strand of the sister chromatid, thus allowing DNA synthesis.

Recently, different groups of researchers have demonstrated that WWOX plays a crucial role in maintaining the integrity of the genome. Indeed, *WWOX* knockout in MEFs exhibits chromosomal alterations and copy number variations [67]. Inhibition of WWOX expression increases the number of DSBs induced by the radiomimetic drug neocarzinostatin in the breast cell line MCF7 [66]. Abu-Odeh et al. showed that WWOX, via its WW1 domain, interacts with ATM (in MCF7 cells) and stimulates its protein kinase activity (in MEF and different breast cell lines) [66]. DSBs lead to the K63-linked polyubiquitination of the lysine 274 of WWOX by the E3 ligase ITCH (E3 ubiquitin-protein ligase itchy homologue), which induces the translocation of WWOX to the nucleus and its association with ATM. Ectopic expression of WWOX in the osteosarcoma U2OS cells induces HR. This research team has provided experimental arguments suggesting that WWOX also promotes repair of DNA single-strand breaks (SSBs) by enhancing the activity of ataxia telangiectasia and Rad3-related protein (ATR) through an ATM-dependent mechanism in different cell lines [104]. WWOX would therefore be a key player in preserving the integrity of the genome by being involved in HR and SSBs.

However, Schrock et al. have shown that WWOX inhibits HR, thus promoting DSB repair by NHEJ in MDA-MB-231 breast cell line [67]. WWOX also inhibits HR in human osteosarcoma U87 cells and Hela cells. Although it has been demonstrated that WWOX has to interact with BRCA1 to inhibit HR, the mechanism by which WWOX acts on this DNA repair mechanism remains largely unknown. The molecular mechanisms by which WWOX maintains the stability of the genome therefore depend on the type of cell. The determination of cells in which WWOX inhibits HR is crucial because it has been demonstrated that enhanced HR in WWOX-deficient cells results in resistance to radiation and cisplatin [67].

The loss of WWOX could therefore lead to genomic instability, which would promote cancer (Figure 3).

## 7. WWOX in the Regulation of Protein Trafficking to the Endosomes, Golgi, and Lysosomes

Interestingly, WWOX binds several proteins playing a role in protein trafficking and in targeting proteins to different subcellular compartments or to lysosomes for degradation [11,70]. Whether these molecular partners, such as SEC23IP (protein transport protein Sec23A-interacting protein), SCAMP3 (secretory carrier-associated membrane protein 3), or LITAF (lipopolysaccharide-induced tumor necrosis factor-alpha factor homologue), also known as SIMPLE (small membrane protein of the lysosome/late endosome), are involved in cancer progression has not been completely elucidated. However, recent findings may indicate that these proteins are cancer-related. For example, SCAMP3 protein was found to be an important regulator of EGFR trafficking and was suggested to play a critical role in EGFR-driven cancers [105]. In the same way, the LITAF protein has been proposed as a tumor suppressor frequently downregulated in several cancer types. The downregulation of LITAF induces cell growth, inhibits apoptosis, and induces cell migration in cancer cells [106]. In addition, the cancer-promoting proteins, TMEM207 (transmembrane protein 207) and VOPP1 (vesicular overexpressed in cancer pro-survival protein 1), found overexpressed in various cancers, bind to WWOX and inhibit its tumor-suppressor activity not by inhibiting its expression but its ability to associate with some protein partners [14,69,107]. It is therefore conceivable that a cell expressing VOPP1, TMEM207, or both, could be cancerous even though it expresses a high level of WWOX [108]. Thus, WWOX may act as an adapter protein with pleiotropic functions resulting from a network of interactions regulating the transcription, the localization, or the degradation of key cancer-related proteins.

## 8. The Function of WWOX in Cancer Development Is Regulated by Its Phosphorylation State

We have seen that the phosphorylation of WWOX on its tyrosine 33 is crucial for its apoptotic activity. Chang et al. recently demonstrated that the phosphorylation of WWOX on its serine 14 also plays a fundamental role in the anticancer function of WWOX. Indeed, melanoma B16F10 cells inoculated into mice metastasize to the lung. pS14-WWOX is expressed in the B16F10 cells infiltrated into this organ, and the injection of Zfra via tail veins prevents B16F10 cells from metastasizing and inhibits the phosphorylation of WWOX on its serine 14 [109,110]. Additionally, the injection of pS14-WWOX7-21 peptide promotes B16F10 cell growth [52,53]. The pS14-WWOX7-21 peptide blocks, while pS14-WWOX antiserum promotes, ceritinib-mediated 4T1 breast cancer stem cells apoptosis [53]. The authors hypothesize that this phosphorylated peptide recapitulates the functional properties of endogenous pS14-WWOX. Altogether, these results strongly suggest that pS14-WWOX promotes cancer cell growth, at least in part, by inhibiting apoptosis. However, the mechanism by which pS14-WWOX acts on cell death remains to be elucidated.

## 9. Conclusions

Numerous scientific studies provide evidence that inhibition or loss of *WWOX* expression can promote cancer in a variety of ways: by inducing cell death resistance, proliferation, migration, invasion, metastasis formation, anaerobic glycolysis, or genome instability. WWOX’s broad spectrum of action is possible because of its ability to modulate many molecular mechanisms through the interaction with multiple protein partners. Most of these molecular mechanisms appear to be cell specific. The tumor suppressor activity of WWOX in a given cell type would therefore be dependent on the availability of one or more of its protein partners. The absence of this availability could explain the existence of cancerous cells expressing high levels of WWOX.

To fully understand the tumor suppressor activity of WWOX, much work needs to be done in order to define all WWOX’s molecular mechanisms and their cell type specificities. Additionally, it has been suggested that the phosphorylation of WWOX on its serine 14 facilitates cancer progression. The research into the presence of this phosphorylated form of WWOX in cancer cells, and the determination of the molecular mechanisms responsible of this phosphorylation, also need to be performed. This work is particularly indispensable for the potential use of WWOX expression as a marker of prognosis and a significant predictor of response to some cancer treatments, such as radiotherapy and chemotherapy.

## Figures and Tables

**Table 1:** Main protein partners involved in the anticarcinogenic molecular functions of WWOX (nd: not defined, P: proline, Y: tyrosine, S: serine, L: leucine, A: alanine, X: any amino acid).

Molecular Partner	WWOX Domain Involved	Partner Domain Involved	Complex Localization	Involved in	Reference
p53	nd	nd	nucleus	apoptosis	[37]
p73	nd	PPPY_487_	cytoplasm	apoptosis	[38]
TRADD	nd	nd	nd	TNF signaling	[55]
TRAF2	nd	nd	nucleus	TNF signaling	[55]
CREB	WW	nd	nucleus	apoptosis	[56]
Hyal-2	WW1	nd	Membrane, nucleus, cytoplasm	cell death	[57,58]
SMAD4	WW1	nd	nucleus	cell death	[57,58]
MEK1	WW and SDR	nd	lysosome	apoptosis	[59]
SMAD3	WW1	PPGY_184_	cytoplasm	TGFβ1 signaling	[34]
Dvl-2	WW1	PPPY_568_	cytoplasm	Wnt signaling	[60]
BCL9-2	WW1	PPPY_561_	nucleus	Wnt signaling	[61]
JAK2	WW1	nd	cytoplasm	JAK2/STAT3 signaling	[15]
STAT3	WW1	nd	cytoplasm	JAK2/STAT3 signaling	[15]
AP-2γ	WW1	PPPY_64_	cytoplasm	transcription	[62]
ERBB4	WW1	PPIY_1037_, PPPY_1285_	cytoplasm	transcription	[63]
C-JUN	WW1	PPPY_64_	cytoplasm	transcription	[64]
RUNX2	WW1	nd	nucleus	transcription	[65]
ATM	WW1	nd	nd	DNA repair response	[66]
BRCA1	WW1	nd	nd	DNA repair response	[67]
ITCH	WW1	LPXY_623_ and LPXY_839_	nucleus	DNA repair response	[8,66]
EZRIN	WW1	PPPPPPPVY_477_	Apical membrane	Signal transduction	[68]
VOPP1	WW1	PPPY_165_	cytoplasm	Protein trafficking/sorting	[14]
TMEM207	nd	PPPY_139_	cytoplasm	Protein trafficking/sorting	[69]
SEC23IP	WW1	PPSY_167_	nd	Protein trafficking/sorting	[11]
SCAMP3	WW1	PPAY_53_ or LPSF_141_	nd	Protein trafficking/sorting	[11]
LITAF	WW1	PPSY_23_	Golgi apparatus	Protein trafficking/sorting	[70]
